# Novel Approaches for Identifying the Molecular Background of Schizophrenia

**DOI:** 10.3390/cells9010246

**Published:** 2020-01-18

**Authors:** Arkadiy K. Golov, Nikolay V. Kondratyev, George P. Kostyuk, Vera E. Golimbet

**Affiliations:** 1 Mental Health Research Center, 34 Kashirskoye shosse, 115522 Moscow, Russian; nikolayquadrat@gmail.com (N.V.K.); golimbet@mail.ru (V.E.G.); 2 Institute of Gene Biology, Russian Academy of Sciences, 34/5 Vavilova Street, 119334 Moscow, Russian; 3 Alekseev Psychiatric Clinical Hospital No. 1, 2 Zagorodnoye shosse, 115191 Moscow, Russian; kgr@yandex.ru

**Keywords:** schizophrenia, GWAS, causal genetic variants, enhancers, brain epigenomics, genome/epigenome editing

## Abstract

Recent advances in psychiatric genetics have led to the discovery of dozens of genomic loci associated with schizophrenia. However, a gap exists between the detection of genetic associations and understanding the underlying molecular mechanisms. This review describes the basic approaches used in the so-called post-GWAS studies to generate biological interpretation of the existing population genetic data, including both molecular (creation and analysis of knockout animals, exploration of the transcriptional effects of common variants in human brain cells) and computational (fine-mapping of causal variability, gene set enrichment analysis, partitioned heritability analysis) methods. The results of the crucial studies, in which these approaches were used to uncover the molecular and neurobiological basis of the disease, are also reported.

## 1. Introduction

Schizophrenia is a severe mental illness that affects between 0.5% and 0.7% of the human population [1]. Both environmental and genetic factors are thought to be involved in its pathogenesis, with genetic factors playing a key role in disease risk, as the heritability of schizophrenia is estimated to be 70–85% [2,3]. According to a current polygenic model of schizophrenia, hundreds of common variants (polymorphisms) are responsible for the bulk of a population’s genetic predisposition [4,5,6]. The additive effect of this common variability can explain up to half of the heritability of schizophrenia measured in twin studies [7,8]. Genome-wide association studies (GWASs) that aim to identify common genetic variants associated with schizophrenia have become key sources of new information regarding the molecular mechanisms that underlie schizophrenia. Many recent studies have been inspired by the idea that the molecular and pathophysiological mechanisms of schizophrenia can be reconstructed based on genetic data [6,9].

**Figure 1:**
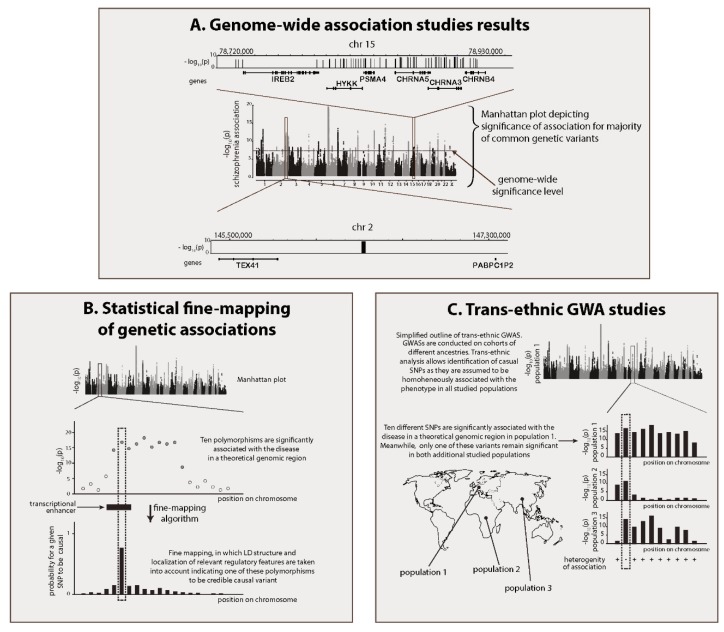

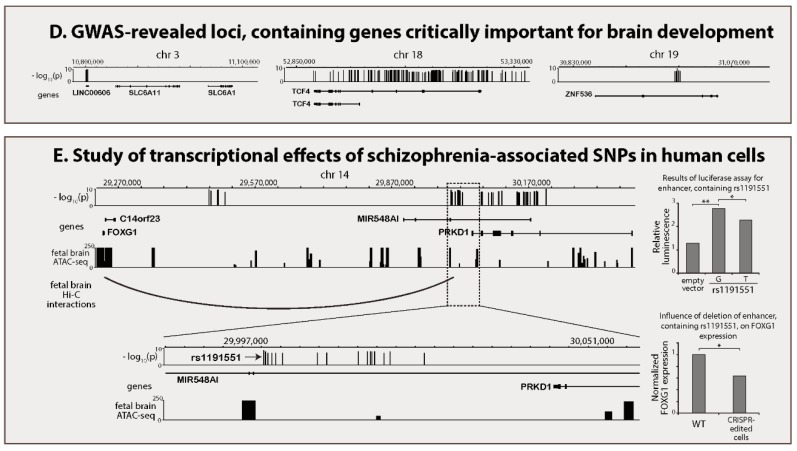
Results of recent schizophrenia genome-wide association studies (GWASs) and basic methods for identification of schizophrenia genes. (**A**) Manhattan plot for recent schizophrenia GWAS meta-analysis [11]. Many GWAS regions cover several genes. However, some of them are located in intergenic DNA. Two representative GWAS regions are zoomed in: significantly associated polymorphisms are depicted as vertical bars. (**B**) Statistical fine-mapping of genetic associations. Each polymorphism’s probability to be causal is assessed in this type of analysis. Additional epigenetic information can improve prediction accuracy. Idealized fine-mapping of GWAS region is depicted. Only one of 10 genome-wide significant polymorphisms (highlighted in dashed rectangle) appears to be credible causal variant. (**C**) Trans-ethnic GWAS. Trans-ethnic study including human populations of three different ancestries is represented. Picture shows one of 10 variants from an idealized GWAS region as consistently (non-heterogeneously) associated with the phenotype. It is assumed that such variants are likely to be causal. (**D**) Study of highly penetrant mutations with brain-related phenotypes. Genes, identified using three different approaches of this class as being schizophrenia genes, are represented. Whole-exome sequencing (WES) studies indicated that rare mutations in *SLC6A1* cause schizophrenia [16]. This strongly suggests that expression of *SLC6A1* is regulated by schizophrenia-associated common variants as one of GWAS regions is located in close vicinity to this gene (200 kb upstream). Rare Mendelian syndrome with psychiatric symptoms confirms role of *TCF4* in schizophrenia development. Various disruptive mutations in this gene lead to dominant autosomal Pitt-Hopkins syndrome, characterized in particular by epilepsy and mental retardation [17]. Finally, phenotypes of model animals with deliberately knocked-out genes identified *ZNF536* as being one of the schizophrenia genes. *ZNF536* double-knockout zebrafish line shows behavioural and neuroanatomical (decreased forebrain volume) changes [18]. (**E**) Description of transcriptional effects of common variation in human neuronal cells. Converging lines of evidence obtained using these methods indicate that *FOXG1* is likely to be regulated by a schizophrenia causal variant. Study of spatial chromatin organization in human fetal brain revealed that one of the schizophrenia GWAS regions interacts with the promoter of *FOXG1* located 750 kb from it [19]. Subsequent study showed that *FOXG1*-interacting SNP rs1191551 is close to one of the fetal brain ATAC-seq peaks [20]. Functional test (luciferase assay) demonstrated enhancer activity of genomic fragment, harbouring rs1191551. Furthermore, this activity was dependent on genotype. The role of this region in regulation of *FOXG1* was additionally confirmed by CRISPR-Cas9 deletion of 500 bp surrounding rs1191551 in neural progenitor cells, with the latter leading to a significant decrease in expression of *FOXG1* but not any other nearby gene.

However, several obstacles exist between identifying significant genetic associations and understanding the biology of this disease. A recent meta-analysis of several GWASs identified more than 150 polymorphisms associated with schizophrenia (Figure 1A) [10,11]. However, each association detected by GWAS, in fact is not an individual polymorphism, but instead represents a group of polymorphisms from the same genomic region. This phenomenon is a reflection of linkage disequilibrium (LD), which describes the co-inheritance of polymorphisms located between two hot spots of meiotic recombination. As a result, these linked polymorphisms could be co-associated with the studied phenotypes [12]. Therefore, each independent association should more accurately be referred to as a genomic locus or region associated with schizophrenia (hereafter referred to as GWAS region). The median length of GWAS regions in the meta-analysis performed by the Psychiatric Genomics Consortium (PGC) is approximately 130 kb, and they can contain several dozens of genes [11]. Likely, only one or a few of the polymorphisms in each region are associated with the disease through a direct causal link [13,14]. GWAS results do not facilitate the direct identification of schizophrenia-associated genes; however, a number of approaches can be used to identify the most plausible target genes of causal variability. Another key obstacle preventing the translation from identifying genetically-associated loci to understanding schizophrenia pathophysiology is the transition from genes to the molecular and cellular systems in which the products of these genes are active. Important tools in this step include the study of brain cell diversity, using single-cell transcriptomics, and analytical approaches that have been developed in the field of systems biology. A new area of research, post-GWAS studies, has been established to address these issues [15]. In this review, we describe the basic approaches that could be utilised to identify schizophrenia genes and to understand schizophrenia pathophysiology, and present the key results of these studies (the concise description of the most widely applied methods is set out in Table 1).

## 2. Identifying Schizophrenia Genes

A number of approaches for identifying the most probable target genes of causal variability exist, including the following: (1) the fine-mapping of causal variability; (2) trans-ethnic GWAS; (3) the study of highly penetrant mutations, leading to neuroanatomical, cognitive, and behavioural changes in humans and animals; and (4) the study of transcriptional regulation in human brain cells.

The association of the whole GWAS region with the phenotype is a consequence of the genetic linkage between a causal variant and other polymorphisms in the region. This simple idea is used in statistical fine-mapping of the causal variance: only those SNPs which are in high LD with all other disease-associated polymorphisms of the region are supposed to be causal (Figure 1B). Furthermore, fine-mapping algorithms consider the types of polymorphisms that are expected to be causal, allowing greater probability weight to be given to nonsynonymous polymorphisms or polymorphisms located inside genomic sites annotated as being regulatory elements. A number of fine-mapping algorithms exist, which utilise different models of genetic association and/or consider the diversity of functional annotations (reviewed in [13]). For example, a fine-mapping analysis could report the Bayesian probability that a tested polymorphism explains the phenotypic association for the entire region. The potential for fine-mapping to identify causal variants depends on the size of the GWAS sample, the number of variants genotyped in the region, and the size of the phenotypic effect–odds ratio (OR)–of this particular variant. Low OR values, which are salient for the variants associated with schizophrenia, are likely to make fine-mapping for schizophrenia challenging, as published attempts to fine-map causal schizophrenia variants have not been successful. However, 25 polymorphisms, including 6 that have been matched to specific genes, have been identified as credible causal variants [11], including the non-synonymous substitution in *SLC39A8*, a variant in the promoter of *ZNF823*, and four polymorphisms in the introns of genes that result in splice variations.

Another population-genetic tool that can be used to narrow down the pool of potential causal variants is trans-ethnic GWAS (Figure 1C) [45]. The first significant success of GWAS for schizophrenia was achieved in a Caucasian cohort [11,46]. Causal variants of schizophrenia are common, which means that, given the universality of disease genetic determinants, the genome regions associated with schizophrenia in other populations should significantly overlap with those identified in Caucasians. At the same time, the LD blocks in other populations have different structures, resulting in only partial overlap between groups of variants associated with schizophrenia in populations with different ancestry, even within the same genomic regions. This intersection of variant sets represents a list of polymorphisms that are genetically linked to the regional causal variant in all analysed populations, and this list will inevitably be shorter than the list of all associated polymorphisms for each population. A GWAS recently conducted by the PGC in an East Asian population has confirmed the effectiveness of this strategy for schizophrenia [29]. A high correlation between the factors that determine genetic predispositions to the disease in European and East Asian populations allowed the two data sets to be merged. A joint analysis showed that trans-ethnic studies can be used to refine association mapping. The approach was also successful in a recent trans-ethnic GWAS that was performed in populations of Latino and African ancestry [30].

If causal variations that slightly alter the expression of target genes in the brain can result in moderate increases in schizophrenia risk, then mutations that radically alter the structures or expression levels of these target genes could have greater penetrance and lead to more severe brain dysfunction [16,47,48]. Several genes, out of hundreds identified within a few hundred kb from GWAS hits (in GWAS region neighbourhoods, hereafter referred to as ‘GWAS RN’) have been associated with monogenic syndromes, with symptoms such as mental retardation, impaired socialization, and epilepsy. These can be considered to be likely schizophrenia-associated genes. For example*, TCF4* haploinsufficiency causes Pitt-Hopkins syndrome, which is characterised by epilepsy and mental retardation (Figure 1D) [17].

Mutations in exon 8 of *CACNA1C*, which encodes a subunit a potential-dependent calcium channel, have been associated with Timothy’s syndrome, characterised by autism [49]. The identification of disease-associated rare mutations can result in conclusions regarding the role played by particular genes from the GWAS RNs in its pathogenesis. Exome sequencing is currently the primary tool used to search for such mutations [50,51]. If exome comparison indicates that mutations in a gene within the GWAS RN can be disease-causing, then the expression of that gene may be influenced by the common causal variant associated with the disease. For schizophrenia, only 3 genes, rare mutations in which significantly increase disease risk have been so far identified, including *SETB1A* [48], *RBM12* [52], and *SLC6A1*, which encodes one of the γ-aminobutyric acid (GABA) transporters [16]. *SLC6A1* is located just near a GWAS region, approximately 200 thousand bp away from its boundary, indicating that the causal variant in this GWAS region likely affects the expression of *SLC6A1* (Figure 1D). The analysis of exomes in patients with autism spectrum disorders was much more fruitful, with a recent large-scale study identifying more than 100 genes with rare disease-causing mutations [53]. Interestingly, these genes are strongly overrepresented in schizophrenia-associated GWAS RNs, suggesting that the presence of rare autism-associated mutations may indicate that a gene located in GWAS RN is schizophrenia gene. The identified group of such genes included *SLC6A1*, *TCF4*, *PRR12*, and a gene for the crucial neuronal transcription factor *FOXP1*.

The high level of structural and functional conservation among protein-coding genes in vertebrates allows animal model phenotypes induced by altered gene expression to act as indicators of links between genes and human diseases, such as schizophrenia. A gene could be considered causal if changes in brain structure or behaviours of a model animal occur as a result of the knockout, knockdown, or overexpression of a gene whose human ortholog is located in schizophrenia GWAS RN. For example, *MEF2C* knockout mice exhibit hyperactivity, repetitive movements, significant learning disabilities, impaired social communication, and other behavioural changes [54], and *NRGN* knockout mice are characterised by impairments in cognitive functions and emotional behaviours [55]. Both these genes are located in schizophrenia GWAS RN. Thus, these phenotypic abnormalities in mice suggest that *MEF2C* and *NRGN* might be schizophrenia genes. The generation of animals with altered gene expression can be performed to validate existing transcriptomic or epigenomic data indicating that this gene may be linked to schizophrenia [21,31]. The overexpression of *TSNARE* and *CNTN4* and the knockout of *FURIN* have been shown to alter the proliferation rate of neuronal precursors and reduce the brain sizes of *Danio rerio* larvae [21]. These genes were located in GWAS RNs and their roles during nervous system development were predicted by human brain transcriptomic data.

Genome editing technologies facilitate the massive creation of knockout animals to systematically test genes from GWAS RNs and examine their roles in the development and normal function of the brain. Recently, zebrafish orthologs of 132 genes located in schizophrenia GWAS RNs were mutated in a large-scale phenotypic screening of mutant *Danio rerio* [18]. Morphological and functional features of the brain, as well as some behavioural patterns, were described in detail for both larvae and adult knockout fish. More than 30 brain-related genes were prioritised as potential schizophrenia causal genes, including *CNNM2*, which encodes a magnesium transporter, a gene encoding the translational repressor GIGYF2, and *ZNF536*, which encodes a transcription factor (Figure 1D). The knockouts of the neuron-specific genes *ZNF804A* and *SNAP91* also led to significant phenotypic changes in *Danio rerio*.

### 2.1. Tissues and Cell Models Used in Studies of Schizophrenia Functional Genetics

Dramatic neuroanatomical, cognitive, and behavioural effects induced by alterations in genes located in GWAS RN can provide reliable evidence to suggest a role for those genes in schizophrenia pathogenesis. In contrast, studies of transcriptional regulation in human brain cells deal with small effects exerted by common variants on the expression of schizophrenia genes, which are much more difficult to interpret. In addition, these small effects are generally tissue-specific; hence, their detection depends on the correct choice of material for study. Therefore, the results obtained using these methods, which we will discuss below, can be used to cautiously infer that a particular gene may be linked to schizophrenia.

Partitioned heritability analysis of schizophrenia GWAS regions have indicated that causal variants are predominantly located not in the coding part of the genome but in active brain enhancers [23,56]. These results have led to the idea that schizophrenia and other multifactorial diseases should be considered to be “enhanceropathies” [57]. According to this model, causal variants located in regulatory sequences affect their affinity to transcription factors, which can lead to quantitative changes in the expression levels of the genes controlled by these enhancers. Although the results of transcriptome comparative analyses have indicated that these changes in RNA expression are very moderate [21,22], small quantitative changes in the transcription of dozens of genes could ultimately result in qualitative functional changes in a patient brain. This model for the molecular mechanisms underlying disease pathogenesis has been reflected by a wide range of functional genomic approaches used to identify schizophrenia genes. These approaches have aimed to identify genes with altered expression patterns in the brain.

Because transcriptional regulation is highly tissue-specific and generally not evolutionarily conserved, the choice of tissue and cell models is of great importance [58,59,60]. The direct interrogation of the human brain is a common approach. Samples are generally isolated from post-mortem cerebral cortex or from brains in various stages of fetal development [19,20]. In addition to primary brain cells, cultured malignant human cells of neural origin can be used. The most popular cell lines are neuroblastomas, including SK-N-SH, SH-SY5Y, and BE (2)-C, as well as NTERA-2 cells, which can be differentiated into neurons [27,61]. There is growing interest to develop cellular models, exploiting the ability of embryonic and induced pluripotent stem cells (iPSCs) to differentiate into neuronal precursors, neurons and glial cells [62,63,64]. In addition, several groups have used cells obtained from nasal biopsies to establish cultured neuronal cells derived from olfactory neuroepithelium (CNON) cultures [65], which facilitates the exploration of primary neurons from both healthy and diseased individuals, without the potential introduction of biases during reprogramming.

Human brain has the most heterogeneous cellular composition among vertebrate organs [66,67,68]. Altered gene expression that results in the development of schizophrenia may occur only in specific cell types. Some studies have indicated predominant roles played by certain cell types in the pathogenesis of schizophrenia, including pyramidal cortical neurons, cortical interneurons, medium spiny neurons of the striatum, and cortical oligodendrocytes [22,34]. iPSCs can be specifically differentiated into these lineages to study the regulation of gene expression in schizophrenia [26,69]. Future research is likely to focus on cell types that are specifically relevant to schizophrenia. In this regard, a rapid development of new protocols for differentiation of iPSCs into specific types of neurons and glia should be mentioned [70].

### 2.2. Massive Transcriptomic Studies and the Comprehensive Mapping of Genomic Regulatory Elements in Brain Cells

Data generated by international collaborations, which systematically describe transcriptomic and epigenomic landscape of human tissues, is particularly important for the identification of schizophrenia genes (see the most significant available sources of such data in Table 2). A complete annotation of transcriptomes and epigenomes of a variety of human cell types is the goal of the several large projects (ENCODE, Roadmap Epigenomics and GTEx) [36,37,39,59,71,72]. However, in all of them, neuronal tissues were underrepresented, and consortia that specifically aim to describe the brain transcriptome in the human population and catalogue brain regulatory sites were necessary. The most prominent such consortia, which were organized subsequently, are CommonMind [40] and PsychEncode [41]. Both of them aim at analysing the influence of common variability on brain transcriptome and epigenome (QTL analysis), cataloguing the remote regulatory elements in various neuronal cells using epigenomic methods (ATAC-seq, DNase-seq, ChIP-seq, Hi-C etc.) and comparisons between the brain transcriptomes of healthy people and those of patients with schizophrenia, bipolar disorder, and autism. Moreover, these consortia systematically compare their results with GWAS data and other reliable genetic information concerning these diseases.

### 2.3. Description of Brain Transcriptomic Landscape

With the development of expression microarrays and RNA sequencing (RNA-seq) technologies, the transcriptome of disease-relevant cell populations has become the easiest to study genome-wide molecular endophenotype of complex traits [73]. To date, two basic approaches exist to identify RNAs whose expression levels are determined by genetic factors associated with schizophrenia. The first is a direct comparison of brain transcriptomes in case-control studies that detects not only differences in the expression of genes directly regulated by causal polymorphisms, but also a lot of secondary transcriptomic changes. These secondary changes can be caused by the direct molecular influences of schizophrenia genes or by environmental changes associated with the disease course, such as the use of psychotropic drugs. Differentially expressed genes from GWAS RNs were initially assumed to be likely schizophrenia genes [21]; however, the latest comparative transcriptome analysis, conducted by PsychEncode, showed that at least 5000 RNAs (approximately one-quarter of all those transcribing in the brain) are differentially expressed in patient brains compared with controls [22]. The vast majority of signals are most likely not genetically determined, and finding out which transcriptional rates among all those that are altered are directly influenced by causal genetic variability is extremely challenging, even considering the position of genes relative to the GWAS regions.

Another approach, which appears to be more productive, utilises the results of genome-wide searches for quantitative trait loci (QTL) that are associated with changes in gene expression (eQTL) or the ratio of RNA isoforms (isoQTL) in the neuronal tissues of healthy people [74,75]. In accordance with the model of schizophrenia as an enhanceropathy, partitioned heritability analysis showed that schizophrenia heritability is enriched in the brain-specific e/isoQTLs [21], indicating that schizophrenia genes should be sought among genes regulated by e/isoQTLs located in GWAS regions. Because the influence of common variability on transcriptional output is relatively small, the number of detected e/isoQTLs depends on the size of the cohort. Large samples of post-mortem brain tissues collected by CommonMind and PsychEncode revealed tens of thousands of e/isoQTLs [21,24]. A number of statistical approaches can be used to test the colocalization of e/isoQTL and GWAS signals, which can favourably distinguish the e/isoQTL analysis from a simple differential transcriptome analysis [76,77]. Only a fraction of e/isoQTLs, that formally fall into GWAS regions, actually colocalize with the GWAS signal. Thus, colocalization analysis can significantly limit the list of potential schizophrenia genes, and despite the discovery of approximately 250,000 e/isoQTL hits in the PsychEncode analysis, only 369 genes are regulated by e/isoQTLs, which actually colocalize with the GWAS signal [24].

Another method for combining the results of e/isoQTL analysis with GWAS data is a transcriptome-wide association study (TWAS), which attempts to predict genetically determined transcriptomic differences between patient and healthy tissues based on the summary statistics from GWAS and e/isoQTL analysis. In contrast with the direct comparisons between e/isoQTL and GWAS hits described above, TWAS considers the phenotypic effects of all polymorphisms, allowing the prediction of differential expression and disease genes at loci that, due to lack of GWAS power, have not yet reached the genome-wide significance level [31,78,79]. Like the e/isoQTL hits, genetic variants that TWAS predict to be associated with expression changes can be tested for colocalization with GWAS signal [76]. The effects of genetic linkage between causal polymorphisms and variants that affect the expression of non-disease genes can be controlled in TWAS using various types of conditional association analysis [22,32]. The largest TWAS for schizophrenia, which was based on the PsychEncode set of brain transcriptomes, identified 62 genes influenced by genetic variants that colocalized with the GWAS signal for schizophrenia. Among those genes are *SNAP91* and *ZNF804A* mentioned earlier as associated with behavioural phenotypes in knockout studies [18,22].

### 2.4. Identification of Brain-Specific Enhancers within GWAS Regions

The transcriptional effects of each of the thousands of non-coding polymorphisms associated with schizophrenia can theoretically be tested in the relevant cell models using functional genomic methods. This strategy has a number of advantages, including the observed changes being free of linkage side effects and the collection of samples from hundreds of people being unnecessary to achieve statistical significance. High-throughput genome editing would allow the precise editing of hundreds of single nucleotide variants in human cells to functionally test all these disease-associated polymorphisms, but it is not yet feasible [80]. The number of variants in GWAS regions with potential functional effects can be reduced by fine-mapping and trans-ethnic GWAS. Unfortunately, these approaches have not yet been productive in the post-GWAS analysis of schizophrenia.

The causal variability of schizophrenia has been reported to be located primarily in the remote regulatory genomic elements [22,25,46,56]. To identify causal variants and their target genes, the pool of functionally tested polymorphisms can be narrowed to those located in neuronal enhancers inside GWAS regions. Active enhancers totally cover a small percentage of the genome and focusing on the effect of the common variants located within them drastically reduces the list of potential causal variants [24,25]. Methods of functional genomics allow the identification of both brain-specific enhancers that contain potential causal variants and genes regulated by those enhancers.

Mass detection of enhancers became possible mainly due to the cataloguing of chromatin marks specific to active remote regulatory elements [81,82]. Widely used universal markers of active enhancers include chromatin openness and the enrichment with certain post-translational histone modifications, especially H3K27ac and H3K4me. The genome-wide mapping of these chromatin marks in neuronal tissues, using DNase-seq, assay for transposase-accessible chromatin using sequencing (ATAC-seq) and chromatin immunoprecipitation sequencing (ChIP-seq), has facilitated the annotation of genomic sites as potential remote regulatory elements that are active in the brain. A comprehensive catalogue of enhancers that are active in schizophrenia-relevant cells is likely to be created soon and would be an important starting point for research programs that aim to identify schizophrenia genes using epigenomic data. Those genes whose expression is influenced by schizophrenia-associated polymorphisms, located within these enhancers, could then be identified using functional techniques. A catalogue of genomic sites tagged with markers of active enhancers for different areas of the brain and various stages of development has been created as part of the Roadmap Epigenomics project [37,59]). The results obtained in PsychEncode and related projects have facilitated the creation of even more detailed genomic regulatory maps of the embryonic brain and adult cerebral cortex. For example, approximately 100,000 potential active enhancers in the prefrontal cortex of adults have been identified, based on ChIP-seq data [24]. Furthermore, ATAC-seq detected more than 100,000 open chromatin sites in cells from the adult prefrontal cortex [25] and more than 50,000 such sites in embryonic cortex [20]. Partitioned heritability analysis has confirmed that these open sites are highly enriched with the causal variability for schizophrenia, indicating that they should be the focus of further functional analysis.

However, the presence of enhancer markers does not guarantee that genomic sites are regulatorily active. A significant part of the genomic sites labelled with these markers are not functionally relevant [83]. Therefore, functional assessments of individual polymorphisms should be preceded by confirmation of enhancer activity for the sites labelled with active markers. Traditionally, methods that rely on the creation of episomal reporter constructs are used for this purpose, and the most common such technique is the luciferase assay. Chromatin context may influence enhancer activity, moreover, the activity of at least some enhancers may be specific for particular target promoters. However, in episome-based methods both factors cannot be taken into consideration [84]; therefore, the luciferase assay is being replaced by functional methods that allow the modification of potential enhancers within the original genomic contexts. These methods use genome (primarily CRISPR-Cas9) and epigenome (CRISPR-dCas9) editing technologies to turn potential enhancers off [85,86]. The assessment of transcriptional changes among genes in the vicinity of a potential enhancer allows both the functional activity of the studied site to be tested and the identification of target genes. Recently, high-throughput functional screening approaches for potential enhancers, based on single-cell RNA-seq technologies and epigenome editing, have been developed to test thousands of genome regions for enhancer activity and to simultaneously identify their target genes in the same experiment [87]. In addition to the classical strategy for enhancer search based on chromatin mark data, an episome-based functional screening approach, known as self-transcribing active regulatory region sequencing (STARR-seq) has been used. This high-throughput method uses specially designed episomal constructs to measure the enhancer activity of all genomic fragments, simultaneously. Originally developed to study the relatively small fruit fly genome [88], STARR-seq was recently adapted to search for enhancers in mammalian genomes [89,90]. This method enables the detection of even those regulatory elements that are not labelled with classical enhancer chromatin marks [88,89].

### 2.5. Prediction of Target Genes for Enhancers Located within GWAS Regions

Genes regulated by brain-specific enhancers from the GWAS regions are not necessarily schizophrenia genes. However, the identification of all genes that are regulated by these enhancers is an important intermediate step prior to proceeding with the large-scale functional verification of individual polymorphisms. The methods utilised for the identification of active enhancers are generally not suitable for application to high-throughput search for their target genes. Although STARR-seq can massively identify active enhancers, it is not designed to identify the target genes of regulatory elements. On the other hand, genomic and epigenome editing methods that are potentially capable of identifying enhancer-promoter pairs remain labour-intensive, and high-throughput versions of these methods are still under development. A number of approaches can be used to indirectly indicate functional relationships between the enhancers from the GWAS regions and specific genes. The results of these indirect methods can often be used as the starting points for further research. The enhancer-gene pairs discovered in these studies are usually the first choices for subsequent functional verifications by genome or epigenome editing [19,26].

The most popular approach for identifying the target genes of potential enhancers from GWAS regions is the analysis of the spatial chromatin organization by C-methods (chromosome conformation capture-based methods), capable to estimate the physical distance between any pair of genomic regions in the nucleus [91]. Although the active enhancers are thought to be spatially close to the promoters of their target genes [81], spatial proximity does not guarantee a functional relationship between an enhancer and a gene [92,93]. Therefore, C-methods do not replace further functional verification. However, high-throughput versions of C-methods, such as Hi-C [94,95], allow the annotation of all target genes for all potential enhancers from GWAS regions in one experiment. These techniques are often used as intermediate step for the detection of genes that may be potentially regulated by enhancers from GWAS regions, before performing the time-consuming functional confirmation.

Recently, several groups described chromatin folding in human neuronal cells and compared this information with genetic data on schizophrenia [19,24,26]. Enhancers from schizophrenia GWAS regions were found to interact with the promoters of a number of genes (*DRD2*, *GRIN2A*, *CACNA1C*, and *FOXP1)*, which have previously been unambiguously linked to schizophrenia. In addition, the promoters of several genes involved in glutamate signalling (*GRIA1*, *NLGN4X*), and genes encoding acetylcholine receptors (*CHRM2*, *CHRM4*, *CHRNA2*, *CHRNA3*, *CHRNA5*, and *CHRNB4*) also interacted with genomic sites that harboured genetic variants associated with schizophrenia. An enhancer that spatially interacts and regulates the activity of the transcription factor FOXG1 gene was discovered in a GWAS region located as far as 750 k bp from *FOXG1* (Figure 1E). Using genome editing, the authors showed that the removal of this fragment in human neural progenitor cells resulted in a significant decrease in *FOXG1* expression. Moreover, the identified enhancer, with the schizophrenia-associated *T* allele in rs1191551, reduced the expression of the reporter gene in the luciferase assay compared with the same enhancer containing the alternative *G* allele. The results of genome-wide mapping of DNA folding in three other neuronal tissues, including the adult prefrontal cortex, iPSC-derived neural progenitor cells, and cortical pyramidal neurons differentiated from neuronal progenitor cells, showed that hundreds of genes interact with enhancers from GWAS regions, with 592 genes identified in the prefrontal cortex, 386 in neural progenitor cells, and 385 in pyramidal neurons. The genome and epigenome editing of several enhancers that spatially interacted with neuron-specific genes in both the embryonic cortex [19] and iPSC-derived neuronal cells [26], confirmed the functional activity of 5 sequences that regulate *ASCL1*, *EFNB1*, *EP300*, *MATR3*, *PCDHA8*, and *PCDHA10*.

The ability to identify promoter-enhancer interactions using Hi-C is highly dependent on the sequencing depth. The reliable mass detection of regulatory loops in human cells requires billions of sequencing reads per standard Hi-C experiment [94,95]. To reduce the cost of deep analysis of promoter-centred interactions (promoter interactome), protocols for enrichment of Hi-C libraries with ligation products between promoters and the rest of the genome were developed [96,97,98,99]. In the near future, methods for analysing the promoter interactome may be applied to neuronal cells.

Attempts are being made to predict enhancer target genes, in silico, using large epigenomic and transcriptomic data sets. The basic principle driving such predictions is the correlation between the activity of an enhancer and a regulated promoter in either different tissues or similar tissues from different individuals. The presence of active histone modifications [100], the level of DNase sensitivity [101], the level of transcription [38,102], or several characteristics at once [103] are used to measure activity in these algorithms. In addition to simple correlation models, controlled machine learning, using a pool of confirmed enhancer-promoter pairs as the training set [103], and module-based joint latent Dirichlet models [58] have also been used. Some algorithms use computationally-predicted binding sites for transcription factors. For example, according to the PsychEncode model, 100,000 enhancers in the prefrontal cortex form approximately 500,000 potential regulatory links with genes. Each link implies that an enhancer located in the same topological domain as the regulated gene contains a binding site for at least one of the 673 analysed transcription factors and that the expression level of this transcription factor correlates with the expression level of the regulated gene in the prefrontal cortex. In this analysis, 13,304 genes were associated with at least one enhancer, including 388 genes that were associated with enhancers located in schizophrenia GWAS regions, which may indicate roles for these genes in the predisposition to the disease [24].

### 2.6. Prioritization of Schizophrenia Candidate Genes in Transcriptomic and Epigenomic Studies

The available information regarding the effects of schizophrenia-associated polymorphisms on the expression of specific genes in the human brain does not unambiguously indicate that the dysregulation of particular genes influences the disease risk. The iso/eQTL and TWAS data can be misleading due to pleiotropic effects, even after testing for colocalization with GWAS signal [76,77,104]. Because most brain iso/eQTL studies were performed on a heterogeneous cell population from the cerebral cortex, many associations between the GWAS signal and gene expression in this complex tissue could be due to pleiotropy. Other epigenomic clues are even less reliable because most of them have not been supported by functional experiments. Neither the spatial proximity measured by C-methods nor the correlations in activity calculated using various sets of epigenomic data can guarantee the influence of an enhancer from the GWAS region on the expression of a potential target gene [87,89]. These methods also fail to guarantee that polymorphisms associated with the disease actually affect the activity of the enhancers, in which the variants are located. Therefore, when using transcriptomic or epigenomic approaches to identify schizophrenia genes, researchers often get a list of candidate genes that contains a significant proportion of false positives.

One way for prioritising these candidate genes is to combine different types of data into a single summary-analysis, such as available large-scale iso/eQTL data on the cerebral cortex [21,22] and epigenomic data from more specific brain cell populations, which are presumably associated with schizophrenia [22,34]. Dozens of specialised software programs have been designed to combine diverse genomic datasets for post-GWAS analyses of polygenic traits, including schizophrenia, to integrate genetic association (GWAS), transcriptomic (iso/eQTL), and epigenomic (ATAC-seq, DNase-seq, ChIP-seq, Hi-C) data (reviewed in [15]).

A joint PsychEncode analysis is an example of such integrative approach. It relied on various strategies to identify potential schizophrenia genes and revealed large, partially overlapping sets of candidates [24]. The locations of schizophrenia-associated variants (promoter and exon polymorphisms) indicated 181 potential candidate genes, whereas the iso/eQTL analysis identified 369 genes, spatial proximity (Hi-C data) analysis indicated 592 genes, and analysis of correlations between expression levels of genes and enhancer-binding transcription factors suggested 388 genes. A total of 1111 genes from the GWAS RNs were identified as potential schizophrenia genes, based on identification by at least one of these methods. Most of these candidates are likely to be false positives; however, those genes that are identified by several independent approaches are considered to be more credible. In the above analyses, 321 genes were identified by at least two of the listed approaches.

## 3. System-Level Data Analysis in Post-GWAS Research

### 3.1. Basic Approaches for Identification of Molecular Networks Associated with a Genetic Predisposition to Schizophrenia

The identification of schizophrenia genes is a key objective of post-GWAS research. However, even an exhaustive list of genes is not sufficient to provide a deep understanding of the disease mechanisms or the subsequent identification of drug targets. Genes must be placed in the context of higher levels of biological organization to determine specific molecular networks, in which the products of schizophrenia-associated genes interact with each other [105,106,107]. The search for such schizophrenia-relevant functional gene sets is performed using approaches developed within the field of systems biology, in which GWAS statistics are combined with various molecular-neurobiological datasets. In general, systemic approaches are independent of the identification of specific schizophrenia genes [108,109]. Therefore, system-level analysis of GWAS data should not be considered a further stage of research that follows the identification of disease genes; instead, systemic research represents an independent strategy that can be used to interpret genetic data.

One of the primary approaches of systemic post-GWAS analysis is the assessment of heritability enrichment among various gene groups. The identification of functional gene groups associated with schizophrenia is performed using a set of specially designed statistical tools: gene set enrichment analysis (GSEA) [109] and partitioned heritability analysis [56,110]. Despite the variety of specific GSEA algorithms available (MAGMA, FORGE, ALLIGATOR, MAGENTA, INRICH), all follow the common scheme. First, on the basis of GWAS data, each individual gene is assigned a figure that reflects the degree of its association with the phenotype. Then, using the statistical tool specific for the particular algorithm, a program evaluates how the association with the phenotype for each gene set differs from the association expected for a random set of genes. Partitioned heritability analysis is an alternative to the GSEA approach that can estimate the proportion of heritability explained by all polymorphisms in the genes of the functional set by calculating the conditional association [56,110].

Functional gene sets for these methods are usually obtained from curated databases and from genome-wide datasets, primarily transcriptomic. Gene Ontology (GO) [43,111], Kyoto Encyclopaedia of Genes and Genome Elements (KEGG) [42,112], and the Mouse Genome Informatics database (MGD) [44,113] are the most commonly used databases for identifying gene functional annotations relevant to schizophrenia (Table 2). Functional gene sets can also be identified by the in-depth analysis of RNA-seq data. Gene network analysis can provide information about groups of functionally related genes from transcriptome sequencing data. The most widely used type of gene network analysis is weighted gene co-expression network analysis (WGCNA), which was designed to detect groups of co-expressed genes based on transcriptome comparisons among different tissues or individuals [33,108,114]. Genes with correlated expression levels form functional groups (modules) that correspond to signalling pathways or molecular cascades. In some modules, the identification of genes whose expression levels can predict the expression levels of other genes is possible. These genes are called nodal or hub genes and are often key regulators of the expression of the remaining genes in the module. Modules detected by WGCNA and gene groups annotated in curated databases can be tested for association with schizophrenia.

A systematic analysis of the association of functional gene sets with schizophrenia, using MAGMA, was conducted as part of the largest GWAS meta-analysis [11]. After multiple comparisons adjustment, 6 schizophrenia-associated functional gene sets were identified, including targets of the translational regulator fragile X mental retardation protein (FMRP) [115], genes encoding proteins of 5-HT_2C_ serotonin receptor complex [116], genes encoding proteins of voltage-gated calcium channels [117], and genes associated with abnormal behaviour, abnormal nervous system electrophysiology, and abnormal long-term potentiation. The largest study of cortical transcriptome, which examined more than 1500 people, combined WGCNA in the human brain with GWAS data for schizophrenia. 90 gene and isoform modules has been identified in this study [22]. Partitioned heritability analysis detected an association with the schizophrenia GWAS signal for 17 modules, in many of which gene expression significantly differed between cases and controls. The discovery of hub genes and GOs that were overrepresented within modules provided insights into their functions. For example, a key hub gene identified in the geneM1/isoM2 module that has been genetically associated with schizophrenia and bipolar disorder is *RBFOX1*, which encodes a neuronal splicing regulator. However, the *RBFOX1* cytoplasmic isoform, which is represented in the geneM1/isoM2 module, is responsible for the translational regulation of several proteins associated with the transmission of excitatory signal in glutamatergic synapses [118]. The geneM7 module was also significantly enriched with schizophrenia GWAS signal. The expression of the geneM7 module is increased in the brains of people with schizophrenia and bipolar disorder. Genes involved in the recycling of synaptic vesicles are significantly overrepresented in this module, and one of the hub genes for this module is the gene encoding the neuronal splicing regulator NOVA2.

A somewhat orthogonal approach for the search of schizophrenia-associated protein subnetworks was suggested by Chang et al. [119]. Authors used PGC2 schizophrenia GWAS summary statistics [35] to calculate gene-level *p*-values, then they mapped nominally significant genes (*p*-value < 0.01) to a human protein–protein interaction (PPI) network constructed based on the iRefindex database [120]. Next, the largest subnetwork, which contains products of genes significantly associated with schizophrenia, was extracted. This subnetwork, named “largest connected component” (LCC), consisted of 402 proteins (nodes) and 620 PPIs (edges), and was significantly larger than randomly generated LCCs, which underline its relevance. Genes of several KEGG pathways were overrepresented in the detected LCC, among which are “synaptic plasticity”, “neural development”, “long-term potentiation”, “neurotrophin signaling pathway”, “ERBB signaling pathway”, “MAPK signaling pathway”, and “T cell receptor signaling pathway”. To further focus on subnetworks relevant to schizophrenia pathogenesis, the authors used the “GWAS edge-based network search” (Gens) algorithm, which is specifically designed for searching for PPI subnetworks enriched in the heritability of the studied disease [121]. Gens analysis consistently indicated a module, which included key N-methyl-D-aspartate receptor (NMDAR) genes DLG1, DLG2, DLG4, ERBB4, GRIN2A, and GRIN2B as the causal schizophrenia molecular network. It is worth noting that genes of NMDAR proteins had long been suggested to be associated with schizophrenia development. Furthermore, this idea was supported by various rare variant studies [122,123]. However, none of the recent post-GWAS analysis, conducted by PGC, confirmed overrepresentation of schizophrenia-associated common variants in this gene set [11,46].

### 3.2. Brain Cell Populations Relevant to Schizophrenia

Hereditability enrichment can be estimated not only for functional gene sets but also for sets of marker genes associated with specific cell types. One of approaches to search for cell subpopulations pathophysiologically relevant to certain diseases is based on this principle. The validity of this approach for the study of schizophrenia is confirmed by the fact that, when analysing markers of various human tissues, it is the marker genes of brain cell populations, which are significantly enriched with SNP-heritability of schizophrenia [124]. In general, genetic data have indicated that the brain is the site where pathophysiological processes associated with schizophrenia take place [25,34,46,124].

However, within the brain, schizophrenia appears to have a very complex cellular and ontogenetic substrate. The systematic annotation of markers associated with various neuronal and glial cell subpopulations has been facilitated by the development of single-cell transcriptomics [66,67]. Partitioned heritability analysis, based on published single-cell transcriptomic data, indicates the existence of several neuronal subpopulations whose marker genes are enriched with schizophrenia heritability [34]. These subpopulations include cortical and hippocampal pyramidal neurons, cortical interneurons, and medium spiny neurons of the striatum. The development of schizophrenia appears to be caused by the distortion of certain molecular processes in these cells. The consolidation of information regarding the gene modules identified as being genetically associated with schizophrenia in the PsychEncode analysis [22] and single-cell transcriptomic data has indicated that the activities of many modules are specific to certain brain cell populations. The manifestation of the disease is apparently linked to populations of cells, whose markers are overrepresented in the modules associated with schizophrenia. Sixteen out of the 17 modules that are genetically associated with schizophrenia are also associated with specific subpopulations of brain cells. Most of these modules are active in either pyramidal or inhibitory cortical neurons, or both [22]. However, some modules are active in glial cells, indicating that glial cells and neurons are both involved in the processes that result in the manifestation of schizophrenia. For example, the geneM2/isoM13 module is active primarily in oligodendrocytes, and the geneM3/isoM1 module is active in astrocytes.

In addition to genes that are specific to certain cellular subpopulations of the brain, active epigenetic marks, which are largely tissue-specific, can also be used to deduce the cytological basis of the disease [20,25,46]. Thus, open chromatin sites in the prefrontal cortex and genomic regions labelled in the cortex with the H3K27ac chromatin mark, are enriched with the heritability of schizophrenia [25,46], whereas the corresponding active genomic sites in the other tissues do not show such an association with the disease. The ATAC-seq study of embryonic cortical cells at 15–17 weeks post-conception showed that the heritability of schizophrenia is enriched within the open chromatin of the developing human brain [20]. Partitioned heritability analysis indicated that open chromatin sites that are specific to germinal zone cells of the embryonic cortex are significantly enriched with heritability. This region primarily consists of cortical pyramidal neuron progenitor cells of the adult brain. Thus, the pathophysiology of schizophrenia is likely to be associated with both prenatal neurogenesis and processes that occur in differentiated brain cells during later stages of development [6].

### 3.3. Genes Encoding Master-Regulators of Molecular Modules Associated with Schizophrenia

Hub genes and other master regulators of functional disease-associated gene sets are of particular interest due to their importance for understanding the structure of subcellular networks disrupted in schizophrenia. The products of these genes are also important as potential therapeutic targets since through these proteins it is possible to exert specific influence on the molecular subsystems associated with schizophrenia.

An example is the fragile X mental retardation protein (FMRP) encoded by *FMR1*. The trinucleotide repeat expansion in this gene is the cause of the fragile X chromosome syndrome, which is usually accompanied by mental retardation. In the brain, FMRP binds to hundreds of mRNA types, many of which are translated locally in neural dendrites. Often this translation is regulated by synaptic activity that is in good agreement with assumed FMRP role in neuroplasticity [125]. Several independent studies have demonstrated a connection between FMRP-regulated genes and the risk of schizophrenia and autism. Apart from schizophrenia SNP-heritability [11], patient-specific rare and de novo mutations are significantly overrepresented in these genes [8,21]. In different types of neurons, FMRP likely binds to the mRNAs of different genes, and further studies could reveal a subclass of FMRP targets from specific neural population associated with the pathogenesis of schizophrenia.

MicroRNA miR-137 is another macromolecule, which has long been a subject of interest for its potential role in the pathogenesis of schizophrenia. It regulates gene expression at the post-transcriptional level and is involved in neurogenesis, neural differentiation, and neuroplasticity [126]. The locus that harbours the gene encoding this miRNA is associated with schizophrenia, according to GWAS [11,46,127]. Several genes, including *CACNA1C*, *TCF4*, *GRIN2A*, and *ZNF804A*, that have been indisputably associated with schizophrenia have been predicted or experimentally confirmed to be miR-137 targets [128,129]. GSEA has revealed a significant association between bioinformatically predicted miR-137 target genes and schizophrenia [46,130]. The miR-137 targets that have been annotated as participating in axonal guidance and ephrin signalling are the most reliably associated with the disease [130].

RBFOX1 is a neuronal protein that has been identified as the key hub of an expression module associated with schizophrenia [22]. The gene encoding RBFOX1 is also genetically associated with the disease [11]. The cytoplasmic isoform of RBFOX1, which is more closely linked to schizophrenia, regulates translation and mRNA stability. By binding to its targets at the 3′end, this isoform activates translation and stabilises mRNA molecules. Studies in mice have shown that many genes regulated that way by RBFOX1 are related to signal transduction at glutamatergic synapses [118,131]. To better understand the function of this expression module, the activity of the RBFOX1 protein must be more comprehensively characterised in human neurons.

Another protein that is thought to control the expression of some schizophrenia-associated genes is the transcription factor TCF4. As mentioned above, the nearby GWAS signal and the fact that rare mutations in this gene cause Pitt-Hopkins syndrome, clearly indicate a role of TCF4 in the pathogenesis of schizophrenia. The search for schizophrenia-associated master regulators in the CommonMind Consortium RNA-seq data and RNA-seq performed on CNON populations both identified TCF4 as a plausible candidate [132]. In recent years, active research has been conducted on brain-specific genes that are regulated by TCF4 to determine how these genes may influence the risk of psychotic disorders, through RNA interference and ChIP-seq studies in several model cell systems, in particular SH-SY5Y neuroblastoma cells, iPSC-derived neuronal precursor cells, and differentiated glutamatergic neurons [61,132,133,134,135]. TCF4 target genes are preferentially expressed in cortical pyramidal neurons and involved in the development of the nervous system, synaptic signal transmission, and ion transport, based on GO-term enrichment analyses [61,135]. Many of the predicted TCF4 targets are also FMRP targets, suggesting that these two functional modules substantially overlap [135]. Although approximately 5000 genes may be targets of TCF4, according to ChIP-seq data, MAGMA shows that this huge gene set is nominally enriched with GWAS signal for schizophrenia [61]. Partitioned heritability analysis indicates that TCF4 binding sites in the genome are enriched with the GWAS signal [135]. Finally, the particular link between *TCF4* and schizophrenia is supported by the overrepresentation of de novo mutations identified in schizophrenia among the group of genes that are regulated by TCF4 [61].

Disease master regulator genes can be genetically associated with a phenotype (as seen for *MIR137*, *RBFOX1*, and *TCF4*), although it is not necessarily true for all of them. Nevertheless, transcription factors, as well as translation and splicing regulators that have been identified by post-GWAS studies as credible schizophrenia genes, such as the neural transcriptional inhibitor ZNF536 [11,18], the transcription factor FOXP1 [53], and the translational repressor GIGYF2 [18], are the priority subjects of further studies examining disease-relevant gene modules. The study of other potential schizophrenia genes, encoding expression regulators and also being hubs of gene modules associated with the disease in WGCNA analysis, also seems promising. An example of such gene is the gene of early response transcription factor EGR1, which is located in a rather large GWAS region that harbours more than a dozen other genes [11]. This protein may play an important role in neuroplasticity [136]. Furthermore, *EGR1* is one of the key hub genes of the gene21/isoM30 module in the PsychEncode WGCNA analysis [22]. This module, which includes other early genes (*ARC*, *NPAS4*, *NR4A1*) and some late-response genes (*BDNF*, *HOMER1*), is nominally associated with schizophrenia [22].

### 3.4. Disease Molecular Modelling as a Path to the Inference of Etiological Mechanisms

Data on gene product molecular interactions can be used in post-GWAS analysis for assessment of heritability enrichment as described in Section 3.1. However, alternatively this information can be incorporated in disease modelling frameworks. In some cases, such complex models, including GWAS data as well as transcriptomic, epigenomic and system-level datasets, can eventually hint at genes and molecular networks underlying disease development.

Some such models are primarily constructed for prediction of disease risk, improving prediction of austere frameworks based solely on genetic data (e.g., simple SNP-based logistic regression). Other models are devised specifically for inference of relevant genes and pathways from GWAS data. The integrative risk gene selector (iRIGS) is an example of the latter type of models [137]. This model can be described as a Bayesian algorithm that integrates multi-omics data and information regarding molecular networks to predict risk genes in GWAS loci and biological processes in which these genes converge. The algorithm selects exactly one gene from each GWAS RN, taking into account transcriptomic and epigenomic evidence that supports association of a given gene with the disease. At the same time, gene choice in iRIGS is influenced by closeness of selected genes in a GO-based molecular network. All genes are attributed with a score that considers molecular evidence pertaining to this specific gene as well as the contribution of this gene to the compactness of a molecular network comprised by all selected genes together. The algorithm is configured to maximise closeness of inferred genes through an iterative process. This piece of iRIGS is based on the notion that causal genes should converge in tight functionally related clusters in such molecular networks. During the first round, scores are assigned to each gene based solely on transcriptomic and epigenomic clues, indicating relevance of the gene to the disease. Then, the disease molecular subnetwork, consisting of only top genes from each GWAS locus, is constructed. One GWAS locus is taken and scores for all genes from this locus are recalculated, taking into account their closeness to other genes in a disease molecular subnetwork. The gene with the highest recalculated score then substitutes for the gene representing this locus in a primary disease molecular subnetwork. This procedure is repeated for each GWAS locus. One could notice that after one round of such network adjustment, gene scores in the first GWAS locus could dramatically change owing to changes in subnetwork composition. That is why the process is repeated until risk genes converge on a stationary distribution.

Wang et al. selected schizophrenia as an example of polygenic disease with poorly understood biology to validate the utility of iRIGS. A total of 108 genome-wide significant loci from PGC2 schizophrenia GWAS [46] as well as brain differential gene expression data [21] and enhancer-promoter links from the fetal brain Hi-C [19] and FANTOM5 project [38,102] were submitted to iRIGs. The algorithm was able to identify 104 high-confidence schizophrenia risk genes (HRGs). These HRG genes turned out to be preferentially expressed in brain-related tissues in prenatal stages of development. Functional analysis showed that HRG were significantly enriched in several biochemically annotated gene sets, among which are FMRP targets, RBFOX1 (GPM6A, MEF2C, KCNC3, TCF4, etc.) and miR-137 (GRIN2A, TCF4, ZNF804A, RORA, CSMD1, etc.) regulated genes, genes of postsynaptic density (PSD) proteins, genes related to the presynaptic active zone, and genes involved in formation of calcium channels and calcium signaling (CACNA1C, CACNB2, PTK2B, GPM6A, etc.). Additionally, comparison of HRGs with brain-related MGI Mammalian Phenotype Ontology (MPO) annotations revealed significant enrichment of HRGs in 33 gene sets after Bonferroni correction. The enriched sets included “abnormal nervous system physiology”, “abnormal nervous system morphology”, “abnormal brain morphology”, and “abnormal behavior”.

A Deep Structured Phenotype Network (DSPN) is an interpretable multilayer deep-learning framework constructed under the PsychEncode project [24]. This model uses both genotype and transcriptomic data as an input and combines a Deep Boltzmann Machine architecture with internode connections derived from a schizophrenia gene regulatory network described in the same paper. DSPN includes several intermediate molecular layers in addition to genotypes and disease status: expression levels of genes, activity of regulatory elements, predefined gene groupings (cell-type marker genes and coexpression modules) and multiple higher layers for inferred groupings (hidden nodes). The primary aim of a DSPN is improvement of phenotype status prediction, and it indeed performs 5.1x better than simple genotype-based linear regression predictor (performance accuracy: 73.6% vs. 54.6%) and 1.8x better than linear regression, which considers both transcriptomic data and genotype (73.6% vs. 63%). However, a crucial advantage of this model is its interpretability: intermediate-level nodes can be prioritized based on their contribution to prediction accuracy, and nodes with the highest rank can be supposed to be highly relevant to disease mechanisms. Thus, 31 out of 5024 brain WGCNA modules were suggested to contribute significantly to schizophrenia status prediction in the DSPN model. Several genes from the schizophrenia GWAS RN belong to these modules, e.g., RBFOX1, C4A, CLU, and NRGN. These modules can be further characterized by enrichment of specific functional gene sets and cell-type marker genes. Among KEGG-derived ontology terms enriched in multiple disease-relevant modules are “spliceosome/mRNA splicing”, “synaptic vesicle cycle”, “chromatin modification”, “calcium signalling”, “Hippo signalling” and several immune-related terms.

### 3.5. Genetically Informed Schizophrenia Categorization and Understanding of Disease Biology

Phenotypic heterogeneity and molecular intricacy have led to the suggestion that schizophrenia as well as many other complex disorders represents superimposition of several etiologically independent entities. On the other hand, for a long time, it was debated that current diagnostic boundaries in psychiatry are imperfect and cases with different diagnoses can actually have the same biological pathway being genetically disturbed, and slightly different environmental factors could coax them into manifestation of apparently different symptoms. Now, it has been firmly established that many psychiatric conditions to a greater or lesser extent share their genetic background with many GWAS loci demonstrating pleiotropic effects on several diseases [138,139]. The most significant genetic correlation of this kind has been shown for schizophrenia and bipolar disorder (r = 0.70 ± 0.02, *p*-value < 1.0 × 10^−6^). Rearrangements in psychiatric disease classification, which include separation of superimposed traits into individual internally homogenous nosological units and aggregation of biologically congenerous but currently separate nosologies, will be advantageous both for patient treatment and further investigation of the biology of brain disorders.

One promising way for such phenotype re-categorization is meta-analysis of genetic data concerning various disease-adjacent traits, which is becoming more and more feasible in the age of publicly available biobank-scale GWAS summary statistics for a plethora of complex traits. Multidimensional datasets, in which each SNP associated with the disease (threshold level of significance for selection of SNPs is more or less arbitrary) can be attributed with the numbers, representing normalized strength of the association between this SNP and dozens of probed traits. Then, these SNPs can be grouped with the help of one or other available clustering algorithms. Discovered clusters can be considered groups of variants contributing to predisposition to different phenotypic disease subtypes, which could potentially correspond to molecular subtypes and eventually be recognized as full-fledged nosological units. These subtypes can be primarily characterized from a phenotypic point of view by traits that contribute most to cluster discrimination, and from a molecular point of view by gene sets enriched in each cluster (genes from GWAS RNs tagged with SNPs from each particular cluster). Biological pathways and cell types revealed by analysis of such disentangled disease molecular subtypes may not be discernible in undeconvoluted schizophrenia GWAS datasets. That is why it is potentially highly beneficial for system-level biological interpretation of GWAS results to conduct studies of this type.

For all we know, such a strategy has not yet been applied to psychiatric diseases. However, papers were published describing similar categorization studies for several other well-characterized polygenic traits, e.g., type 2 diabetes (T2D) [140,141].

In Udler et al. clustering of 94 T2D-associated GWAS variants by their influence on 47 metabolic traits using Bayesian non-negative matrix factorization (bNMF) produced five clusters of genetic loci. Each described grouping was characterised by a specific pattern of association with traits used for the clustering. Two clusters were associated with reduced beta-cell function, differing from each other by proinsulin level (high and low), which can indicate that they represent defective insulin processing and defective insulin synthesis, respectively. Loci, harbouring many well-established beta-cell specific T2D genes, were assigned to these two clusters, including MTNR1B, HHEX, TCF7L2, SLC30A8, HNF1A, HNF1B, ARAP1 and SPRY2. The three other clusters were associated with different types of insulin-resistance: obesity-mediated (high BMI and waist circumference), “lipodystrophy-like” fat distribution (low BMI, adiponectin, HDL-cholesterol, and high triglycerides), and disturbed liver lipid metabolism (low-serum triglycerides). Top loci in obesity-mediated insulin-resistance cluster were the well-known obesity-associated loci FTO and MC4R. Three of the top four weighted loci in disturbed liver lipid metabolism cluster, GCKR, CILP2/TM6SF2, and PNPLA3, have been previously reported to be associated with non-alcoholic fatty liver disease [142].

In-depth molecular characterization of these groupings revealed that each cluster was enriched with enhancers and promoters active in specific tissues. This enrichment was consistent with disease mechanisms suspected to underlie each cluster. For example, the defective insulin-processing cluster was significantly enriched with regulatory elements active in pancreatic islet cells, with adipose-specific active chromatin overrepresented in a “lipodystrophy-like” fat distribution cluster, whereas the disrupted liver lipid metabolism cluster was enriched with regulatory elements active in hepatocytes.

In another study of T2D [141], a different clustering approach, referred to as C-means clustering, was exploited. Surprisingly, whereas a slightly different set of loci, not exactly the same set of traits, and another clustering method were used, five of six clusters detected in this study broadly matched five clusters described by Udler et al. Unfortunately, none of the T2D studies reported whether the revealed clusters were enriched with genes of specific physiological processes or cellular pathways. However, two independent methods of clustering T2D variant-trait associations resulted in rather robust loci groupings, thereby indicating potential biological relevance of the described disease subtypes. Therefore, the application of similar approaches in post-GWAS exploration of psychiatric conditions appears exceptionally promising.

Despite the fact that this approach to SNP clustering, leveraging results of multiple GWAS for disease categorization, has not yet been applied to schizophrenia, some alternative methods were used to identify multidimensional non-allelic genetic interactions [28,143,144]. Owing to the lack of statistical power, such studies are oftentimes restricted by interactions of several candidate genes and therefore are prone to “winner’s curse” and publication bias. Work published by Arnedo et al. claimed to discover “eight classes of schizophrenia” exploiting nonnegative matrix factorization of genotyped subjects and SNPs [145]. Although this study gained a tremendous level of public attention, it was severely criticized by recognized experts in the field of psychiatric genetics for methodological flaws and an overall dearth of statistical evidence [146].

## 4. Conclusions

Successful deciphering of the human genome, followed by a sharp drop in sequencing costs during the last decade, has resulted in real breakthrough in psychiatric genetics [6,9]. Although speculative concepts regarding the genetic underpinnings of schizophrenia long dominated this field, we finally have access to an abundance of reliable and reproducible data regarding genetic associations with the disease. The study of the molecular basis of the disease process can now safely rely on this solid foundation. However, despite the enormous body of work performed by large consortia and independent research groups, how these genetic factors result in observable changes in the brain functions of patients with schizophrenia remains unclear. The lack of a clear concept describing the molecular basis underlying the pathogenesis of schizophrenia is particularly reflected by the stagnation of research aimed at identifying new drugs [147]. The current status of molecular genetics of schizophrenia is especially disheartening when compared with the achievements being made in the study of other common diseases, such as diabetes mellitus type 2 or Crohn’s disease, for which the genetic substrates and pathogenetic mechanisms were almost unknown in the mid-2000s [148,149]. Progress toward understanding the molecular mechanisms of schizophrenia may be impeded by several factors, including the excessively high polygenicity of schizophrenia, poor knowledge of cell diversity in the human brain, difficulties creating relevant cell models, and a lack of information regarding the molecular biology of individual brain cell populations. The research areas likely to transform the field in the coming years, include the following: (1) large-scale multi-ethnic GWAS that facilitate the fine-mapping of schizophrenia causal genetic variants; (2) single-cell transcriptomics, to create an exhaustive catalogue of human brain cell subpopulations, analogous to that produced for the mouse brain [66], as a thorough knowledge of the cellular diversity of the brain will facilitate the discovery of a set of specific cell types involved in the pathogenesis of schizophrenia; (3) protocols for the differentiation of human induced or embryonic pluripotent stem cells into specific brain cell populations; (4) detailed molecular descriptions of developed high-quality cellular models, including the mapping of candidate enhancers using standard epigenomic techniques (ATAC-seq, ChIP-seq); and (5) new methods that combine the functional verification of enhancers with the identification of target genes, based on multiple genome/epigenome editing and the detection of transcriptional changes in millions of individual cells [87]. Thus, the discovery of functional enhancers that contain potential causal variants and the identification of their target genes will likely be greatly simplified by scientific advances. Finally, the increasing simplicity and precision of CRISPR-Cas systems will allow the testing of individual polymorphism effects on gene expression to become a routine laboratory procedure [80,150]. This approach may become the gold standard for the confirmation of functional relevance for putative causal variants.

The rapid development of these new research areas suggests that the next decade of research may lead to a qualitative leap in our understanding of the molecular mechanisms underlying schizophrenia. With a very high probability, the conceptualization of psychotic disorders at the molecular level will result in radical changes in medical practice. Nosology and diagnostics can undergo profound transformation. Such a leap in the understanding of schizophrenia would also facilitate the development of effective drug therapies for schizophrenia, in addition to the development of measures that aim to prevent the disease manifestation in people at risk [151,152].

## Figures and Tables

**Table 1:** Approaches most commonly used in schizophrenia post-GWAS studies.

Molecular Biology Techniques			
Method	Description	Application in Post-GWAS Studies	Selected Publications
RNA-seq	High-throughput sequencing of reverse-transcribed RNA allows quantitative assessment of transcriptional activity for each gene in a given sample. This technology made the most basic molecular phenotype easily measurable.	Direct case-control comparison of brain RNA-seq datasets is used for the search of genes with altered expression (see differential expression analysis). Being joined with genome-wide genotyping data for a human cohort, brain RNA-seq datasets can be used in e/isoQTL analysis. Alternatively, populational RNA-seq data is necessary for construction of brain-specific WGCNA networks, which are then used in gene set enrichment analysis or heritability enrichment analysis.	[21,22]
ChIP-seq	This method is essentially chromatin immunoprecipitation (IP) coupled with high-throughput sequencing. It is employed for genome-wide search for DNA sites occupied by proteins of interest. Antibodies against chromatin-interacting proteins of interest are incubated with sheared chromatin, and DNA bound by antibodies is precipitated, purified, and subjected to sequencing.	The approach is used for genome-wide annotation of sequences potentially acting as enhancers. IP with antibodies against enhancer-specific histone modifications (e.g., H3K27ac and H3K4me1) are especially useful. As causal polymorphisms are expected to localize within enhancers, ChIP-seq-predicted neuronal enhancers harbouring schizophrenia-associated SNPs are primary targets for subsequent functional interrogation with luciferase test and genome/epigenome editing. Alternatively, given a high level of overall enhancer tissue specificity, enhancers annotated in the cell type can be used in heritability enrichment analysis to test the relevance of this cell type for disease development.	[23,24]
Chromatin accessibility assays (DNase-seq and ATAC-seq)	The techniques are based on enrichment of sequencing libraries with histone-depleted genomic regions. This is achieved by means of enzymes specifically targeting such sites in chromatin (DNase I and Tn5 transposase) with subsequent preferential amplification of short DNA fragments excised by these enzymes. Accessible chromatin-enriched libraries are subjected to next-generation sequencing.	It is widely assumed that TSS-distal open chromatin regions are colocalized with active enhancers, thus these methods along with ChIP-seq are used for enhancer inference. As causal polymorphisms are expected to localize within enhancers, DNase-seq / ATAC-seq-predicted neuronal enhancers harbouring schizophrenia-associated SNPs are primary targets for subsequent functional interrogation with luciferase test and genome/epigenome editing. Alternatively, given a high level of overall enhancer tissue specificity, enhancers annotated in the cell type can be used in heritability enrichment analysis to test the relevance of this cell type for disease development.	[20,25]
High-throughput proximity ligation assays (Hi-C and Promoter Capture Hi-C)	In high-throughput proximity ligation methods, distances between pairs of genomic sites are assessed by means of proximity ligation followed by next-generation sequencing. Hi-C allows measurement of proximity between any pair of genomic sites. Promoter Capture Hi-C offers the opportunity to assess distances between promoters and any other genomic site with reduced sequencing burden compared to Hi-C.	It is believed that promoters spatially interact with their cognate enhancers. Thus, proximity-ligation methods are utilized to infer functional enhancer-promoter links. If an enhancer, involved in the enhancer-gene loop in neuronal cells, at the same time harbour schizophrenia-associated SNPs, this physical proximity can be utilized as evidence for genes having a causal role in the disease. Functional links between this gene and the enhancer, containing a schizophrenia-associated genetic variant, can be accurately confirmed with genome/epigenome editing.	[19,26]
Episome-based functional reporter assays (luciferase assay and STARR-seq)	A potential enhancer sequence is inserted in specially-designed episome, harbouring reporter genes. Transfection of the construct into cells with subsequent measurement of reporter gene expression levels allows assessment of enhancer activity for a tested sequence in a given cell type. The luciferase test was designed for low-throughput testing of enhancer sequences (one at a time), whereas the STARR-seq allows testing of thousands of genomic sites in one experiment.	The luciferase assay is used to confirm regulatory activity of schizophrenia-associated genomic sites predicted to be enhancers in brain cells. Often, such predictions are based on the results of the aforementioned epigenomic methods: ChIP-seq, chromatin accessibility assays or high-throughput proximity ligation assays. Besides that, the influence of alternative alleles of schizophrenia-associated SNPs on activity of enhancers, in which given polymorphic sites reside, can be measured with the luciferase assay. STARR-seq can potentially be used to probe all genomic sites on their enhancer activity in a given brain cell type. Localisation of schizophrenia-associated variants inside STARR-seq confirmed brain enhancers can be considered strong evidence of causality.	[19,27]
Genome editing (CRISPR-Cas)	In situ targeted manipulation of genomic sequence exploiting bacterial Cas DNA nuclease (e.g., Cas9) guided by short RNA fragments (gRNA). The currently available CRISPR-Cas tools allow in some cases single nucleotide-precision genome editing and therefore creating isogenic models for functional testing of SNPs. Other CRISPR-Cas systems are used to excise short fragments (several hundreds of bp) of DNA from the genome.	CRISPR-Cas approaches can be used in human neural cells for substitution of individual schizophrenia-associated nucleotides or excision of entire enhancers, harbouring such nucleotides. These enhancers are usually predicted with ChIP-seq or/and chromatin accessibility assays. Editing is followed by assessment of changes in expression of enhancer cognate genes. Alternatively, genome editing is used for creation of knock-out model animals to test the role of potential schizophrenia genes in brain development and function.	[18,19]
Epigenome editing (CRISPRi)	These tools were designed for targeted in situ epigenetic inactivation of regulatory sequences in the genome. This was made possible by abolishing nuclease activity of Cas9 and fusion of this protein with various eukaryotic transcription inhibitory domains (e.g., KRAB-domain, MECP2 inhibitory domain).	Epigenome editing is used as a simplified alternative of genome editing for functional confirmation of regulatory activity of enhancers containing schizophrenia-associated polymorphisms. Besides that, CRISPRi can be used in the search for genes regulated by such enhancers.	[26,28]
**Computational Methods**			
Statistical fine-mapping of genetic associations (BIMBAM, CAVIAR, FINEMAP, etc.)	This approach is represented by a family of instruments that seeks to determine causal variants in each GWAS region. Basically, fine-mapping algorithms seek to predict which polymorphism in a disease-associated linkage disequilibrium (LD) block better explains association of the entire region with the phenotype.	In some cases, causal SNPs can be confidently identified within schizophrenia GWAS regions with statistical fine-mapping. If such variants are localized outside of the coding regions, their position relative to predicted and functionally confirmed brain enhancers can be assessed. Episome-based functional reporter assays and genome/epigenome editing can be subsequently applied to confirm enhancer activity and find genes controlled by this particular schizophrenia-associated enhancer.	[11,28]
Trans-ethnic GWAS meta-analysis	In trans-ethnic GWASs, results of several GWAS experiments, obtained for genetically distant populations, are compared side-by-side. This approach is based on the notion that true causal variants must be associated with the disease in any studied cohort, independent of background LD structure. Thus, trans-ethnic GWASs take advantage of differences in LD structure among various human populations to fine-map causal polymorphisms.	All strategies described for statistical fine-mapping of genetic associations are applicable to trans-ethnic GWASs.	[29,30]
Differential expression (DE) analysis	There are a number of computational tools for decent comparison of RNA-seq (or expression microarray) results between different tissues, different experimental conditions or individuals with different phenotypes. Collectively these tools can be referred to as DE analysis. The main output of DE analysis is a list of genes, of which expression significantly differs between compared datasets.	Genes differentially expressed in brains of cases and controls could be potentially involved in schizophrenia development. However, it is extremely hard to pinpoint truly causal genes among thousands of genes found to be differentially expressed in these two cohorts. In recent years, this strategy has been largely replaced by transcriptome-wide association studies and iso/eQTL analysis.	[21,22]
e/isoQTL analysis	Joined analysis of RNA-seq data and matched genome-wide genotyping results obtained from the cohort of individuals allows discovery of relationships between SNPs and levels of gene expression in the studied tissue. SNPs that significantly influence levels of expression or splicing pattern of any gene are called eQTLs and isoQTLs.	Originally, it was assumed that SNPs associated with schizophrenia, and at the same time being brain e/isoQTLs, are highly likely causal variants. Furthermore, genes regulated by such SNPs in the brain are credible schizophrenia genes. However, accumulation of data regarding e/isoQTL in the human brain (now thousands of such SNPs are detected) has led to the notion that e/isoQTL can co-localize with disease-associated variants by chance. Therefore, more rigorous approaches are now utilised to reliably confirm colocalization of GWAS and e/isoQTL signal (see “Colocalization tests”).	[21,22]
Transcriptome-wide association study (TWAS)	Joined analysis of GWAS summary statistics and e/isoQTL analysis summary statistics makes possible inference of genetically-determined differences in expression levels of all genes between cases and controls of GWAS study in a given tissue (which is the tissue used in e/isoQTL analysis). The output of TWAS is a list of genes, of which expression significantly differs between cases and controls.	TWASs, based on schizophrenia GWASs and e/isoQTL analysis of human neuronal tissues, predict genes regulated by schizophrenia-associated polymorphisms. Essentially, there is definition of schizophrenia causal genes. However, owing to the phenomenon of LD, some TWAS-detected genes can be controlled by polymorphisms linked to causal ones. To account for these artefacts, additional tests, confirming colocalization of GWAS and e/isoQTL signals, are usually conducted (see “Colocalization tests”).	[22,31,32]
Colocalization tests (SMR/HEIDI, Sherlock, coloc, etc.)	Colocalization tests are statistical tools used to verify whether association of a given polymorphism with two different phenotypes (e.g., disease and level of RNA of a specific gene in eQTL analysis) are based on the LD between two different causal SNPs or actual pleiotropy of one genetic variant.	Colocalization tests are often employed to confirm colocalization of a schizophrenia GWAS signal and signal from neuronal e/isoQTL analysis. This same approach is used both in simple e/isoQTL analysis and in TWASs. Given the rapid growth of both GWAS and e/isoQTL datasets, the peril of random colocalization of signals increase, which can subsequently lead to false-positive schizophrenia genes. Therefore, relevance of colocalization tests in these approaches has been realized in recent years.	[22,31,32]
Weighted gene co-expression network analysis (WGCNA)	WGCNA is a data-driven method used for extraction of information, regarding gene sets, from expression data. In WGCNA, a number of RNA-seq (or expression microarray) datasets from the same tissue of different individuals is analysed. Alternatively, in some cases, information from various tissues can be used. Correlations in expression of all possible gene pairs are calculated, then correlation-based clustering of genes is performed. Clusters (modules) of tightly correlated (co-expressed) genes are assumed to represent biologically meaningful gene sets.	Modules detected with WGCNA analysis in human brains are useful gene sets, which are widely used in gene set enrichment analysis and partitioned heritability analysis. These methods allow detection of WGCNA modules relevant to schizophrenia development.	[22,33]
Gene set enrichment analysis (MAGMA, FORGE, ALLIGATOR, MAGENTA, INRICH)	Gene set enrichment analysis (GSEA) is a toolbox of algorithms (e.g., MAGMA, FORGE, ALLIGATOR, MAGENTA, INRICH) used for inference of causal disease gene sets from GWAS summary statistics. Basically, gene-level p-values of disease association are calculated with these algorithms. Then, a list of studied gene sets and genes falling in each of these gene sets are submitted to the algorithm. Association of each gene set is assessed, based on gene-level p-values. Gene sets which survive multiple comparison adjustments are considered to be disease-relevant. Gene sets used in GSEA can be derived from various sources: curated databases (see Table 2), WGCNA analysis, experimentally defined gene sets (e.g., genes regulated by certain transcription factors, miRNA, or RNA-binding molecules) or markers of different cell types.	Various GSEA algorithms are used in schizophrenia post-GWAS studies to detect disease-relevant molecular networks and cell types. Among the most commonly used gene sets in this kind of analysis are: brain-derived WGCNA modules, genes specifically expressed in various cell populations, gene sets associated with neurological and behavioural changes in mice (from MGD database, see Table 2), and experimentally compiled gene sets with some pre-existing evidence, indicating their association with the disease (e.g., FMRP targets, RBFOX1 targets, genes of proteins of NMDA receptor complex, etc.).	[11,34]
Partitioned heritability analysis	Partitioned heritability analysis is an alternative means to GSEA to detect phenotype-relevant gene sets or any other subset of genomic regions (ChIP-seq or ATAC-seq peaks, introns, exons, etc.). Heritability explained by certain types of genomic regions is compared in this algorithm with heritability explained by randomly sampled genomic regions. Regions significantly enriched in GWAS-derived disease heritability are assumed to be disease-relevant. All remarks about gene sets used in GSEA are applicable to partitioned heritability analysis.	All strategies described for gene set enrichment analysis are applicable to partitioned heritability analysis. Additionally, enhancer markers (derived from ChIP-seq or/and chromatin accessibility assays) for various tissues can be used to infer schizophrenia-relevant cell types.	[20,22,34]

**Table 2:** Databases and other valuable datasets widely used in schizophrenia post-GWAS studies.

Resource	Type of Information	Description	Link
Psychiatric genomics consortium (PGC)	GWAS results	Data on PGC-conducted GWASs for schizophrenia and various other common psychiatric diseases. Summary statistics are publicly available.	[35]
MRC centre for neuropsychiatric genetics and genomics	GWAS results	Publicly available summary statistics of the largest published meta-analysis of schizophrenia GWASs.	[10]
ENCODE (Encyclopedia of DNA elements)	Epigenomic and transcriptomic datasets, regulatory annotations	Raw and processed data on gene expression and chromatin structure in various human and mouse cell types. Integrative annotation of regulatory elements in dozens of cell types is also available. All datasets are publicly accessible.	[36]
Roadmap Epigenomics project	Epigenomic and transcriptomic datasets	Raw and processed data on gene expression and chromatin structure in human stem cells and primary ex vivo tissues. All datasets are publicly available.	[37]
FANTOM5 (Functional annotation of the mammalian genome)	Transcriptomic datasets, regulatory annotations	Сomprehensive data on RNA expression in different mammalian cell types. Annotations of promoters, enhancers and promoter-enhancer links are compiled. All datasets are publicly available.	[38]
GTEx (the genotype-tissue expression project)	Transcriptomic datasets	Genome-wide expression profiles for 54 non-diseased tissues of a human body.	[39]
CommonMind consortium knowledge portal	Genotype data, epigenomic and transcriptomic datasets	Expression data with matched genotype and ATAC-seq data from hundreds of postmortem brain samples from donors with schizophrenia, bipolar disease, and individuals with no neuropsychiatric disorders. Access to raw data is controlled. Results of differential expression and eQTL analysis are publicly available.	[40]
PsychENCODE consortium knowledge portal	Genotype data, epigenomic and transcriptomic datasets, system-level integrative models	Epigenomic and transcriptomic datasets from hundreds of brain samples from donors with psychiatric conditions and individuals with no neuropsychiatric diagnosis on different ontogenetic stages. Raw data is access-controlled. Outputs of various types of follow-up analysis (eQTL, TWAS, WGCNA, cell type-specific regulatory networks, etc.) are publicly available.	[41]
KEGG (Kyoto encyclopedia of genes and genomes) pathways database	Collection of annotated gene sets	Publicly available curated functional gene sets.	[42]
GO (gene ontology) database	Collection of annotated gene sets	Publicly available lists of genes annotated by GO consortium as sharing “molecular function”, residing in the same “cellular component” or participating in the same “biological process”.	[43]
MGD (mouse genome informatics database)	Collection of annotated gene sets	Gene sets compiled by MGD, based on the comprehensive catalogue of mouse mutations and phenotypes caused by these mutations.	[44]
